# Propolis Suppresses UV-Induced Photoaging in Human Skin through Directly Targeting Phosphoinositide 3-Kinase

**DOI:** 10.3390/nu12123790

**Published:** 2020-12-10

**Authors:** Da Hyun Kim, Joong-Hyuck Auh, Jeongyeon Oh, Seungpyo Hong, Sungbin Choi, Eun Ju Shin, Soon Ok Woo, Tae-Gyu Lim, Sanguine Byun

**Affiliations:** 1 Department of Biotechnology, Yonsei University, Seoul 03722, Korea; dahyun.kim@yonsei.ac.kr (D.H.K.); ojo1661@naver.com (J.O.); 2 Department of Food Science and Technology, Chung-Ang University, Anseong 456-756, Korea; jhauh@cau.ac.kr; 3 Korea Food Research Institute, Wanju 55365, Korea; hsp@kfri.re.kr (S.H.); sej296@naver.com (E.J.S.); 4 Division of Bioengineering, Incheon National University, Incheon 22012, Korea; 201921148@inu.ac.kr; 5 Department of Agricultural Biology, National Institute of Agricultural Science, Rural Development Administration, Wanju 55365, Korea; wooso1@korea.kr; 6 Department of Food Science & Biotechnology, Sejong University, Seoul 05006, Korea; tglim@sejong.ac.kr

**Keywords:** propolis, skin, matrix metalloproteinase-1, UV, phosphoinositide 3-kinase

## Abstract

Propolis is a resinous substance generated by bees using materials from various plant sources. It has been known to exhibit diverse bioactivities including anti-oxidative, anti-microbial, anti-inflammatory, and anti-cancer effects. However, the direct molecular target of propolis and its therapeutic potential against skin aging in humans is not fully understood. Herein, we investigated the effect of propolis on ultraviolet (UV)-mediated skin aging and its underlying molecular mechanism. Propolis suppressed UV-induced matrix metalloproteinase (MMP)-1 production in human dermal fibroblasts. More importantly, propolis treatment reduced UV-induced MMP-1 expression and blocked collagen degradation in human skin tissues, suggesting that the anti-skin-aging activity of propolis can be recapitulated in clinically relevant conditions. While propolis treatment did not display any noticeable effects against extracellular signal-regulated kinase (ERK), p38, and c-jun *N*-terminal kinase (JNK) pathways, propolis exerted significant inhibitory activity specifically against phosphorylations of phosphoinositide-dependent protein kinase-1 (PDK1) and protein kinase B (Akt). Kinase assay results demonstrated that propolis can directly suppress phosphoinositide 3-kinase (PI3K) activity, with preferential selectivity towards PI3K with p110α and p110δ catalytic subunits over other kinases. The content of active compounds was quantified, and among the compounds identified from the propolis extract, caffeic acid phenethyl ester, quercetin, and apigenin were shown to attenuate PI3K activity. These results demonstrate that propolis shows anti-skin-aging effects through direct inhibition of PI3K activity.

## 1. Introduction

Ultraviolet (UV) radiation causes skin photoaging, which is characterized by wrinkle formation, dyspigmentation, and increased fragility [1]. Reduction in collagen and elastic fibers is the major contributing factor for wrinkling of the skin [2]. UV radiation induces signal transduction pathways that lead to the transactivation of matrix metalloproteinases (MMPs), which are enzymes responsible for the degradation of extracellular matrix proteins [3]. MMP-1 in particular is known to majorly contribute in the UV-mediated collagen degradation process causing skin wrinkle formation [4]. As degradation of collagen is known to be the primary reason for UV-mediated skin aging, blocking MMP-1 activity or expression has been recognized as a promising strategy for preventing skin aging [2].

Phosphoinositide 3-kinases (PI3Ks) are lipid kinases that produce phosphatidylinositol 3,4,5-trisphosphate (PIP3) by phosphorylating phosphatidylinositol 4, 5-bisphosphate (PI 4,5-P2). PIP3 can initiate signaling events leading to the activation of phosphoinositide-dependent protein kinase-1 (PDK1) and protein kinase B (Akt) [5]. PI3K and its downstream PDK1 and Akt pathways have been known to play a crucial role in maintaining homeostasis, and abnormal regulation of these signaling pathways are implicated in various pathological conditions, including cancer, immunological disorders, metabolic syndrome, and aging [6]. Importantly, activation of PI3K is also reported to participate in the process of skin aging [7]. As PI3K can promote the expression of MMP-1 leading to collagen breakdown [8], targeting PI3K can provide a therapeutic window to effectively regulate MMP-1 levels and prevent skin aging.

Propolis is produced by bees using substances collected from various parts of plants. Multiple studies have identified the constituents of propolis, which belong to diverse groups of compounds: flavonoids, terpenoids, phenylpropanoids, stilbenes, lignans, sugars, and amino acids [9,10]. Propolis extracts have been known to have applications for the treatment of various diseases due to their anti-oxidant, anti-inflammatory, anti-bacterial, anti-diabetic, and anti-cancer effects [9,11,12], and thus have been widely used as nutraceutical ingredients. However, the effect of propolis against photoaging in human skin is not fully understood, and its molecular target is largely unknown. Therefore, in the current study, we sought to investigate the protective effects of propolis against UV-induced skin aging using human skin tissues and elucidate the molecular mechanism and active components responsible. 

## 2. Materials and Methods 

### 2.1. Reagents

Crude propolis was purchased from a local market in Chungju, Korea. Antibodies to detect phosphorylated extracellular signal-regulated kinase (ERK) 1/2 (Thr202/Tyr204), phosphorylated c-jun *N*-terminal kinase (JNK) (Thr183/Tyr185), phosphorylated p38 (Thr180/Tyr182), phosphorylated PDK1 (Ser241), phosphorylated Akt (Thr308), and total Akt were purchased from Cell Signaling Biotechnology (Beverly, MA, USA). Antibodies against total ERK, total JNK, total p38, and vinculin were obtained from Santa Cruz Biotechnology (Dallas, TX, USA). Antibody for MMP-1 was obtained from R&D systems (Minneapolis, MN, USA). The reference standards of apigenin, caffeic acid, caffeic acid phenethyl ester (CAPE), ferulic acid, gallic acid, naringenin, and quercetin for high-performance liquid chromatography (HPLC) analysis were purchased from Sigma-Aldrich (St. Louis, MO, USA).

### 2.2. Extraction of Propolis

One gram of propolis was immersed in 10 mL of 80% EtOH and extracted by stirring for 48 h. The extracted propolis was filtered through a non-woven fabric and a filter paper (Watmann # 2). The extracts were then subjected to a 0.22 μm filter and concentrated. 

### 2.3. Cell Culture and UV Irradiation

Hs68 human dermal fibroblast cells were purchased from the American Type Culture Collection (ATCC, Manassas, VA, USA). Cells were cultured in Dulbecco’s modified Eagle’s medium (DMEM) (Corning Inc., Corning, NY, USA) containing 10% fetal bovine serum (FBS) (Sigma, St. Louis, MO, USA) and 1% penicillin/streptomycin (Corning Inc., New York, NY, USA) at 37 °C in a 5% CO_2_ incubator. Cells were irradiated using a UVB cross-linker (6 × 8 W, 312 nm, Model Bio-link BLX, Vilber lourmat, Paris, France). The dosage which produced the highest MMP-1 level without cytotoxicity was chosen as the optimal UVB irradiating condition.

### 2.4. Excised Human Skin and UV Irradiation

Human skin tissues were purchased from Biopredic International (Rennes, France). The human skin tissues had been obtained following the French Law L.1245-2 CSP. Biopredic International has been approved by the French Ministry of Higher Education and Research for the acquisition, transformation, sale, and export of human biological material to be used in research (AC-2013-1754). This study complied with all principles set forth in the Helsinki Declaration. Human skin tissues were incubated with DMEM containing 10% fetal bovine serum with penicillin/streptomycin in a 5% CO_2_ incubator at 37 °C. Ex vivo human skin tissues were treated with propolis extracts (PPE) and exposed to UV once a day for 10 days. 

### 2.5. Cell Cytotoxicity Assay

Cells were seeded in 96-well plates and incubated for 24 h before being cultured in serum-free DMEM. The indicated concentration of PPE was added, and 48 h after the treatment, cell cytotoxicity was measured using the CellTox™ Green Cytotoxicity Assay (Promega, Madison, WI, USA). The fluorescence was measured using the Varioskan multimode microplate reader (Thermo Fisher Scientific, Waltham, MA, USA).

### 2.6. Enzyme-Linked Immunosorbent Assay (ELISA)

Cells were seeded in 6-well plates at a density of 1.8 × 10^5^ cells/well. When cells reached 80–90% confluence, the medium was replaced with serum-free DMEM. Cells were treated with PPE (2.5, 5, 10 and 20 µg/mL) and incubated for 1 h. After UVB irradiation (0.03 J/cm^2^), cells were incubated for 48 h. Media were centrifuged and supernatants were collected and stored at −80 °C. The concentration of MMP-1 protein in the medium was determined using human total MMP-1 DuoSet ELISA kits (R&D systems Inc., Minneapolis, MN, USA), according to the manufacturer’s protocol. The absorbance was measured using the Varioskan multimode microplate reader (Thermo Fisher Scientific, Waltham, MA, USA).

### 2.7. Quantitative Real-Time PCR

Total RNA was extracted from cells using a NucleoSpin RNA isolation kit (Macherey-Nagel, Düren, Germany). RNA purity and concentration were determined by measuring the absorbance at both 260 and 280 nm. cDNA was synthesized from 0.5 µg of total RNA using a ReverTra Ace^®^ qPCR RT Master Mix with gDNA Remover (TOYOBO, Osaka, Japan). MMP-1 mRNA expression levels were analyzed using real-time PCR with Step One Plus Real-Time PCR system (Thermo Fisher Scientific, Waltham, MA, USA) and THUNDERBIRD^®^ SYBR^®^ qPCR Mix (TOYOBO, Osaka, Japan). Primers used in the reaction were MMP-1 (Forward, GCATATCGATGCTGCTCTTTC; Reverse, ACTTTGTGGCCAATTCCAGG) and Glyceraldehyde-3-Phosphate Dehydrogenase (GAPDH) (FW, CCATCACCATCTTCCAGGAG; RV, ACAGTCTTCTGGGTGGCAGT). 

### 2.8. Histological Analysis

Formalin-fixed tissue samples were embedded in paraffin, and 3~4 µm sections were cut. Tissue sections were cut on glass slides, de-paraffinized with xylene, and rehydrated through a series of graded ethanol baths. The sections were then stained with a Masson’s trichrome satin kit (Sigma, St. Louis, MO, USA). After the washing step, slides were dehydrated and cleared in xylene before mounting. Stained slides were then photographed using a light microscope.

### 2.9. Immunoblot Assay

Cells were lysed with RIPA lysis buffer containing a protease and a phosphatase inhibitor cocktail (Sigma-Aldrich, St. Louis, MO, USA). The concentrations of protein in the lysates were determined using a Pierce BCA Protein Assay Kit (Thermo Fisher Scientific, Waltham, MA, USA). Samples were separated using 10% sodium dodecyl sulfate-polyacrylamide gel electrophoresis (SDS-PAGE) gels with 5% stacking gels and transferred to a nitrocellulose (NC) membrane (PALL Corporation, Port Washington, NY, USA). After transfer, the NC membranes were incubated with the specific primary antibodies at 4 °C overnight. Horseradish peroxidase (HRP)-conjugated immunoglobulin G (IgG) was used as a secondary antibody. The protein expression levels were visualized using the ChemiDocTM XRS + System (Biorad, Hercules, CA, USA) or film.

### 2.10. High-Performance Liquid Chromatography (HPLC) Analysis

Dionex Ultimate 3000 HPLC system (Thermo Fisher Scientific, Waltham, MA, USA) and INNO C-18 Column (250 mm × 4.6 mm, 5 μm, Youngjinbiochrom, Seongnam, Korea) were used to analyze the chemical composition of propolis. The mobile phase consisted of 0.3% trifluoroacetic acid (Buffer A) and acetonitrile (Buffer B), which were applied in gradient elution as follows: 0–1 min, 5% B; 1–35 min, 5–35% B; 35–50 min, 35–100% B; 50–55 min, 100% B; 55–56 min, 100–5% B; 56–60 min, 5% B at a flow rate of 0.8 mL/min. The injection volume was kept at 10 μL, and the oven temperature was 45 °C. The compounds detected were identified by comparing with reference standards, and the detection wavelengths were 280 and 340 nm (190–400 nm diode array detector (DAD) scanning.

### 2.11. Kinase Assay

The SelectScreen Kinase Profiling Service (Thermo Fisher Scientific, Waltham, MA, USA) was used to examine kinase activities.

### 2.12. Modeling the Structures of Chemical Compounds on PI3K

Five highest-resolution crystal structures of human PI3K, p110α catalytic subunit, were retrieved from the Protein Data Bank (PDB ID: 6PYS, 4JPS, 5DXT, 4WAF, 5UBR) [13]. In addition, the structures of porcine PI3K, p110γ catalytic subunit, co-crystallized with ATP (PDB ID: 1E8X) and quercetin (PDB ID: 1E8W) were retrieved from PDB [14]. All the structures were superimposed to that of the human PI3K (PDB ID: 6PYS) before further analysis. The structures of CAPE, quercetin, apigenin, gallic acid, caffeic acid, ferulic acid, and naringenin were retrieved from the PubChem database with PubChem compound identifiers of 5281787, 5280343, 5280443, 370, 689043, 445858, and 932, respectively [15]. The structure of PI3Ks and chemical compounds were converted into the PDBQT format using the prepare_receptor4.py and prepare_ligand4.py scripts of the AutoDock Tools, respectively [16].

The chemical compounds were docked onto the PI3K structures using AutoDock Vina (Version 1.1.2) [17]. The docking simulations were executed within a cubic box with size 20 Å. The box was centered at the coordinate of the N9 atom of the ATP molecule, which is located near the center of the ATP. During the docking simulation, up to 20 docking poses were retrieved for each PI3K and chemical compound pair. The docking structures were inspected on the PyMOL Molecular Graphics System, Version 2.5.0 (Schrödinger, LLC, New York, NY, USA).

## 3. Results

### 3.1. Propolis Extract Suppresses UV-Mediated MMP-1 Expression and Collagen Degradation in Human Skin

**Figure 1:**
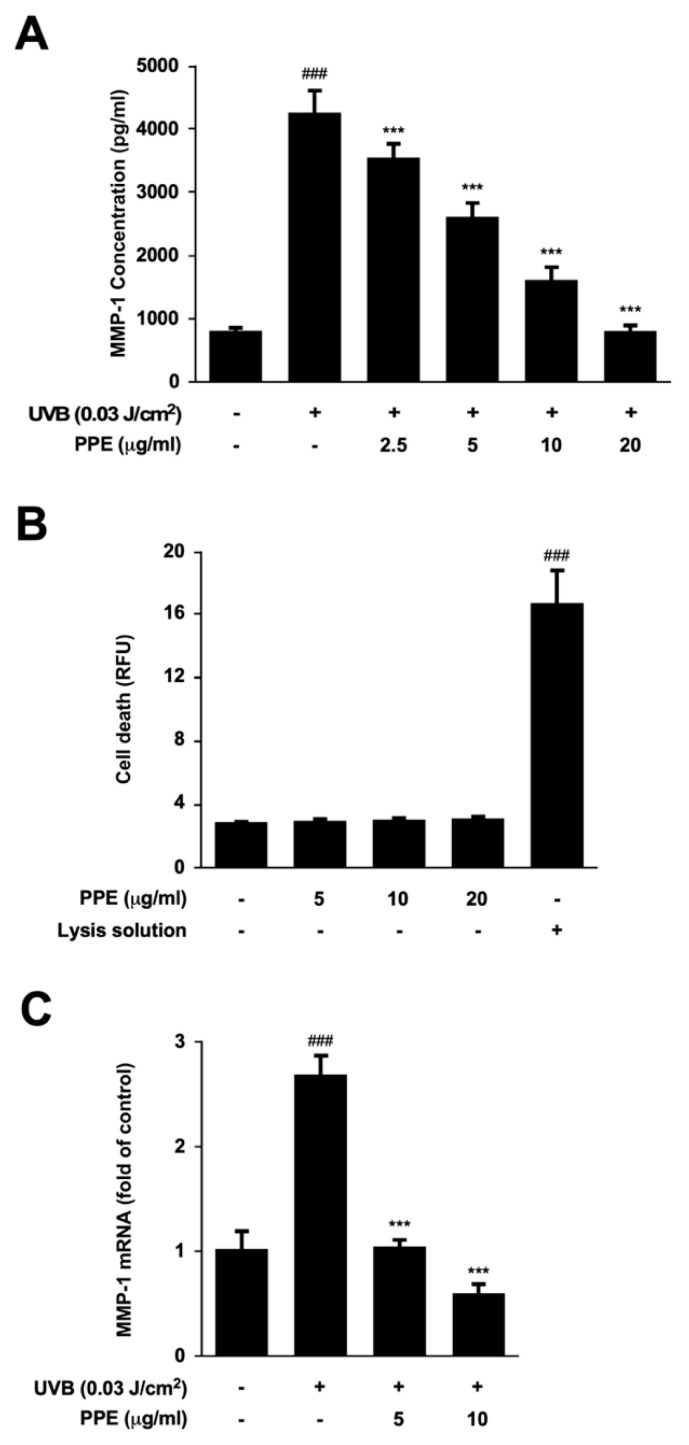
Effect of propolis extract on UV-induced matrix metalloproteinase (MMP)-1 expression in human dermal fibroblasts (HDFs). (**A**) Cytotoxicity of propolis extracts (PPE) was measured using the CellTox Green Cytotoxicity Assay kit. Lysis solution was used as a positive control for cell death; (**B**) Hs68 cells were pre-treated with various concentrations of PPE for 1 h before UVB irradiation (0.03 J/cm^2^). Forty-eight hours after UVB exposure, media was collected and MMP-1 levels in the media were determined by ELISA; (**C**) MMP-1 mRNA levels were examined by RT-PCR. Data are shown as mean ± S.D. ### *p* < 0.001 compared with untreated control; *** *p* < 0.001 compared with the group of UVB exposure alone.

In order to examine the preventive potential of propolis against skin aging, we examined the effect of propolis on UV-induced MMP-1 levels in Hs68 human dermal fibroblasts (HDFs). Propolis treatment displayed significant inhibitory activity against UV-induced MMP-1 production in HDFs (Figure 1A). Notably, propolis extract did not show cytotoxicity in HDFs at the concentrations tested (Figure 1B). Next, the effect of propolis on mRNA expression of MMP-1 was examined. Propolis completely blocked UV-induced MMP-1 mRNA levels, indicating that the propolis-mediated reduction in MMP-1 levels is due to transcriptional control of MMP-1 expression (Figure 1C).

**Figure 2:**
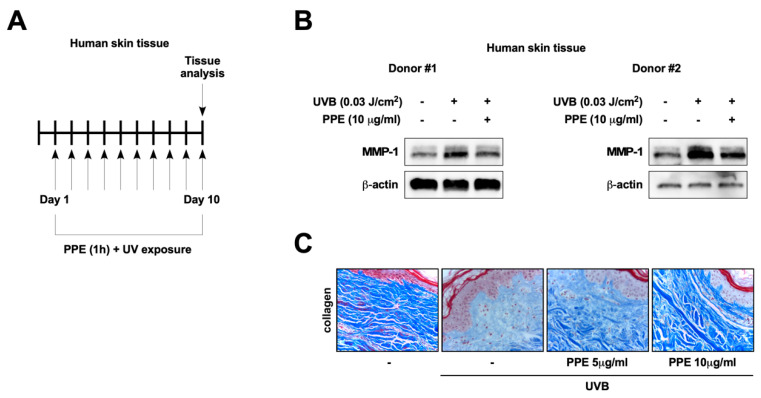
Propolis extract prevents UV-induced matrix metalloproteinase (MMP)-1 expression and collagen degradation in human skin tissues. (**A**) Human skin tissues from two donors were treated with propolis extracts (PPE) and exposed to UVB for 10 consecutive days. At the end of the study, the tissues were fixed in formalin or lysed with lysis buffer for protein extraction; (**B**) MMP-1 expression in human skin tissue lysates was assessed by immunoblotting; (**C**) Collagen fibers were detected with Masson’s trichrome staining.

To further confirm the efficacy of propolis against skin aging, we applied propolis extract on skin tissues from two different donors. We irradiated human skin tissues with UV for 10 days and investigated the protective effect of propolis (Figure 2A). Propolis treatment effectively suppressed UV-induced MMP-1 expression in human skin measured by immunoblotting of MMP-1 (Figure 2B). In addition, propolis prevented UV-induced collagen degradation in human skin tissues (Figure 2C), suggesting that the inhibitory activity of propolis against skin aging can be observed not only in human skin cells, but also in actual human skin tissues. These results demonstrate that propolis can display anti-skin-aging effects at physiological conditions.

### 3.2. Propolis Specifically Downregulates Akt and PDK1 Signaling Pathways

**Figure 3:**
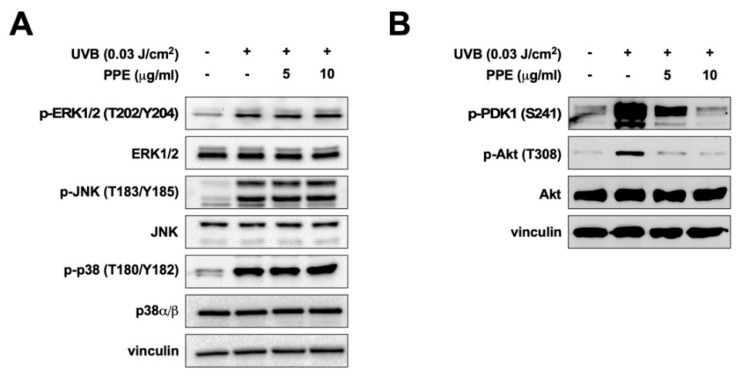
Effect of propolis extract on UV-induced signaling pathway in human dermal fibroblasts. Hs68 cells were pre-treated with propolis extracts (PPE) for 1 h before UVB stimulation (0.03 J/cm^2^). Immunoblot analysis of phosphorylated and total (**A**) extracellular signal-regulated kinase (ERK), c-jun *N*-terminal kinase (JNK), and p38; (**B**) phosphoinositide-dependent protein kinase-1 (PDK1) and protein kinase B (Akt) expression in cell lysates. Vinculin was used as a loading control.

As propolis reduced UV-mediated MMP-1 mRNA levels (Figure 1C), we next examined the effect of propolis on upstream regulators of MMP-1 expression. The MAPKs, including ERK, JNK, and p38, are well-known major players in the UV-mediated signal transduction pathway contributing to skin aging [18]. However, treatment of propolis extract did not show any noticeable effect against UV-induced phosphorylations of ERK, JNK, and p38 (Figure 3A). Phosphoinositide 3-kinase (PI3K) and Akt have also been recognized to mediate UV-driven photoaging [8]. Interestingly, propolis extract exerted a significant inhibitory effect against UV-induced phosphorylation of Akt (Figure 3B). PI3K activates PDK1, which in turn phosphorylates Akt (Thr 308) [19]. Therefore, we questioned if propolis would also affect PDK1 activation. Propolis extract dose-dependently inhibited UV-induced phosphorylation of PDK1 (Figure 3B), suggesting that propolis extract could be targeting the PI3K-PDK1-Akt signaling axis.

### 3.3. Propolis Directly Targets Phosphoinositide 3-Kinase (PI3K)

**Figure 4:**
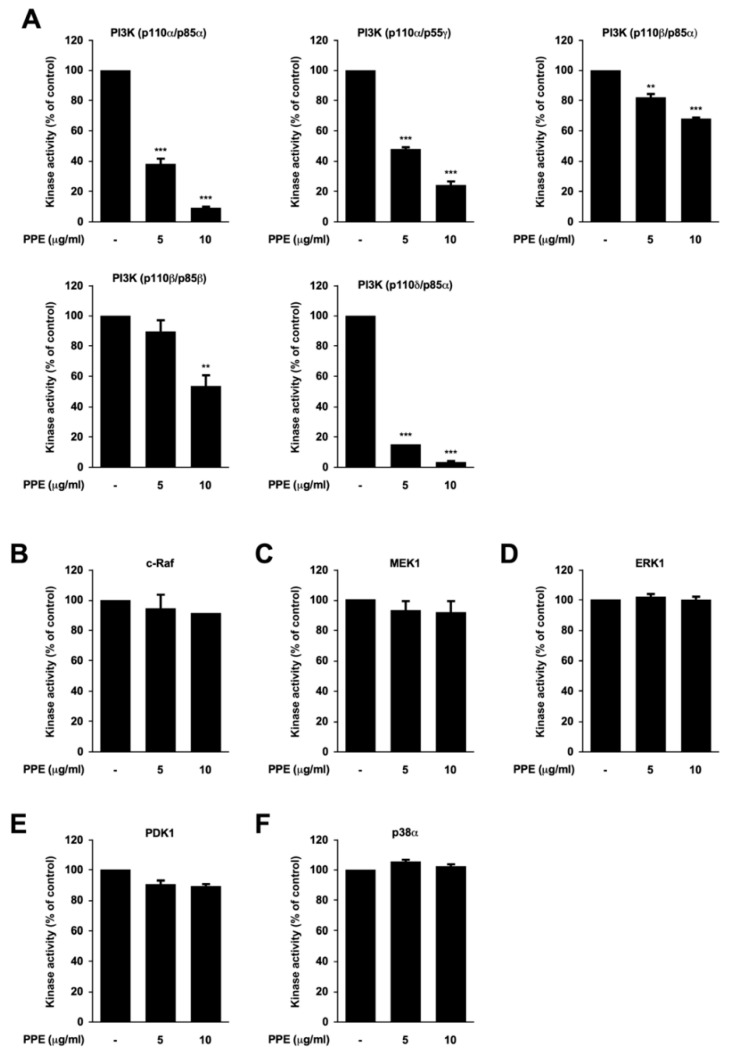
Propolis extract selectively attenuates phosphoinositide 3-kinase (PI3K) activity. Kinase activity of (**A**) PI3K isoforms; (**B**–**F**) c-Raf, mitogen-activated protein kinase (MAPK)/ERK kinase 1 (MEK1), extracellular signal-regulated kinase 1 (ERK1), phosphoinositide-dependent protein kinase-1 (PDK1), and p38α are shown after propolis extracts (PPE) treatment at the indicated concentrations. Data are shown as mean ± S.D. ** *p* < 0.01, *** *p* < 0.001 compared with untreated control.

Since propolis downregulated phospho-PDK1 and phospho-Akt, we examined the effect of propolis extract against PI3K activity. Kinase assay results against a panel of five PI3K isoforms demonstrated that propolis extract can directly inhibit PI3K activity (Figure 4A). PI3Ks containing the catalytic subunit p110α or p110δ were the most sensitive PI3K isoforms towards propolis. To further confirm the specificity of propolis against PI3K, we assessed the effect of propolis extract against several other kinases. Propolis displayed little or no effect against c-Raf, MEK1, ERK1, PDK1, and p38 kinase activity (Figure 4B–F). Collectively, these results indicate that propolis is a selective and potent inhibitory agent against PI3K.

### 3.4. Caffeic Acid Phenethyl Ester (CAPE), Quercetin, and Apigenin Are the Bioactive Components of Propolis in Targeting PI3K

**Figure 5:**
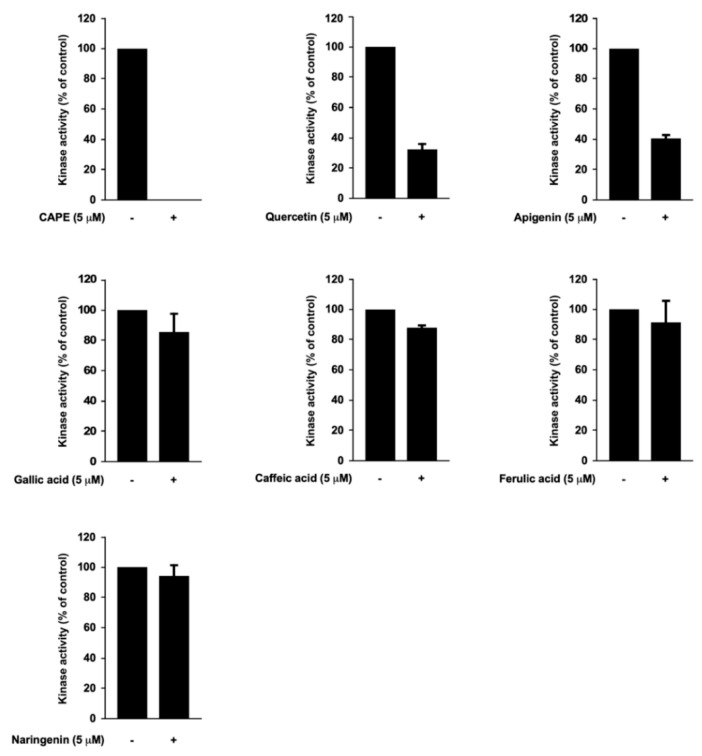
Differential inhibitory activity of propolis compounds against phosphoinositide 3-kinase (PI3K). Effects of caffeic acid phenethyl ester (CAPE), quercetin, apigenin, gallic acid, caffeic acid, ferulic acid, and naringenin on PI3K activity are shown. All compounds were treated at 5 μM. Data are shown as mean ± S.D.

In order to identify the active components responsible for the marked suppression of PI3K activity, we analyzed the chemical composition of propolis. The amounts of apigenin, caffeic acid, CAPE, ferulic acid, gallic acid, naringenin, and quercetin were quantified by HPLC analysis (Table 1). We tested each compound individually against PI3K. CAPE, quercetin, and apigenin showed strong inhibitory effects against PI3K activity, whereas the other compounds displayed relatively minor effects on PI3K activity (Figure 5). As propolis was able to selectively suppress PI3K activity (Figure 4A), CAPE, quercetin, and apigenin appear to be major contributing components to the bioactivity of propolis.

## 4. Discussion

In the current study, we have discovered that propolis exhibits protective effects against UV-induced skin aging. Notably, the anti-skin-aging efficacy of propolis was also confirmed in ex vivo human skin tissues, suggesting that propolis has the potential to exert its effects when applied to human subjects. These results provide a rationale to further develop propolis as a main ingredient for skin products designed to prevent photoaging.

Analysis of the molecular mechanism of propolis led to the identification of PI3K as a direct target of propolis. PI3K has been reported to regulate MMP-1 expression and skin aging [7,8]. UVB activates the PI3K/Akt signaling pathway, which induces MMP-1 in human dermal fibroblasts [8]. Propolis was able to downregulate UVB-induced PDK1 and Akt activation, in addition to directly attenuating PI3K activity. To validate the specificity, we also tested the inhibitory effect of propolis against the activity of several other kinases involved in UV signaling. Propolis appears to selectively inhibit PI3K activity compared to other kinases. In addition, based on our results, we believe that propolis has a preference for PI3Ks containing the catalytic subunit p110α or p110δ. Previous reports have shown that PI3K can display different functions in an isoform-specific manner [20,21]. Moreover, structural analysis on the catalytic subunits of PI3K demonstrates that each isoform has distinct structural features [5]. Thus, the differential inhibitory effects observed from propolis against PI3K isoforms may be attributed to the unique structure of each isoform. 

**Figure 6:**
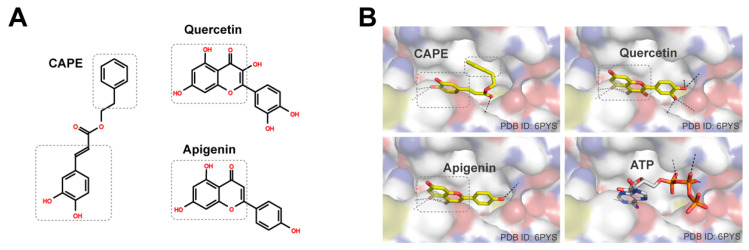
Structures of bioactive compounds and their structural models on phosphoinositide 3-kinase (PI3K) (p110α catalytic subunit). (**A**) The 2D structures of caffeic acid phenethyl ester (CAPE), quercetin, and apigenin. The moieties interacting with PI3K were highlighted with dashed boxes; (**B**) The structural models of CAPE, quercetin, and apigenin on PI3K (p110α catalytic subunit). ATP was located on human PI3K by superimposing the structure of porcine PI3K co-crystallized with ATP to the human PI3K and displaying its ATP part.

Flavonoids have been recognized as major active ingredients in various plants with health-promoting effects [22,23]. Due to their structural features, multiple studies have reported that flavonoids can directly target kinases [24]. In our study, we investigated the PI3K inhibitory potency of flavonoids that were detected in the propolis extract. Interestingly, among the tested compounds, CAPE, quercetin, and apigenin attenuated PI3K activity. To further understand how CAPE, quercetin, and apigenin modulated the activity of PI3K, we have built structural models of these compounds on PI3K. Quercetin and apigenin have the benzopyran moiety, and CAPE has the phenyl moiety conjugated with the adjacent alkene moiety (Figure 6A). These moieties form planar structures that resemble the adenine moiety of ATP. Molecular docking simulations located these moieties at the hydrophobic cleft of PI3K, and their polar atoms can form hydrogen bonds with PI3K polar atoms deep in the cleft, similar to the interaction observed between ATP and PI3K (Figure 6B). In addition, the structural models showed that the phenyl group of CAPE can interact with the nearby hydrophobic surface of PI3K, and the phenyl group of quercetin and apigenin can be placed at the hydrophobic cleft. Quercetin had been successfully co-crystallized with porcine PI3K, and its derivatives were developed as PI3K inhibitors [14]. Collectively, it appears that CAPE, quercetin, and apigenin directly bind to the p110 submit of PI3K to modulate its kinase activity.

Although other compounds from propolis displayed structural similarities with CAPE, quercetin, or apigenin, they exhibited minimal inhibitory effects against PI3K activity. Computational modeling suggested that gallic acid, caffeic acid, and ferulic acid may bind to the PI3K ATP binding site, but with reduced binding affinity (Figure S1). They have relatively small sizes and may not be able to bind strongly enough to compete with ATP. Naringenin is structurally similar to quercetin and apigenin, and it can be modeled to bind to the ATP binding site (Figure S1). However, it does not have a benzopyran moiety, and the pyran ring is reduced. This change is likely to abolish the conjugated planar structure and reduce its binding affinity to PI3K, which could explain its lack of effect against PI3K.

Previous reports have suggested the involvement of some of the active compounds in regulating PI3K or MMP-1. Apigenin was reported to suppress UVA-mediated MMP-1 expression in the HaCaT cell line and promote collagen synthesis in dermal fibroblasts [25,26]. Previous studies suggested that CAPE and quercetin can reduce UV-induced MMP-1 expression in cells and skin tissues [27,28]. Apigenin and quercetin have been shown to suppress PI3K activity [29,30]. Based on these findings, we propose that CAPE, quercetin, and apigenin may function as major contributing factors for the anti-skin-aging and PI3K inhibitory effects exerted by propolis. 

**Figure 7:**
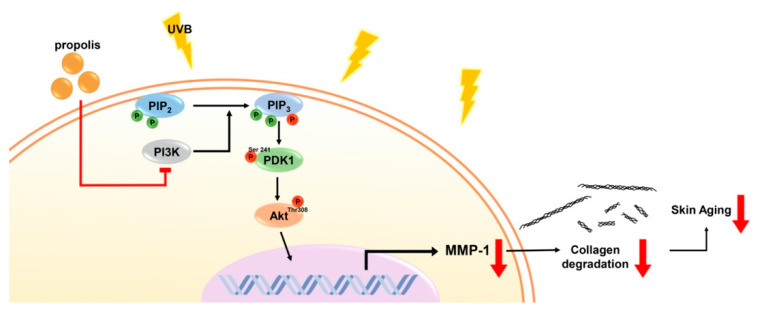
Schematic diagram summarizing the molecular mechanism of propolis. PIP_2_, Phosphatidylinositol 4,5-bisphosphate; PIP_3_, Phosphatidylinositol (3,4,5)-trisphosphate; PI3K, phosphoinositide 3-kinase; PDK1, phosphoinositide-dependent protein kinase-1; Akt, protein kinase B; MMP-1, matrix metalloproteinase-1.

In the current study, we have found that propolis can show anti-skin-aging effects in human skin tissues, suggesting that the anti-skin-aging activity of propolis can be exhibited in physiological conditions. More importantly, we have identified that propolis directly suppresses the kinase activity of phosphoinositide 3-kinase (PI3K) and its downstream signaling pathway (Figure 7). Discovering PI3K as the molecular target and identifying active components of propolis will aid in expanding the therapeutic applications of propolis. 

## Figures and Tables

**Table 1:** Chemical composition of propolis extract identified by HPLC.

No.	Compound	mg/kg of Extract
1	Gallic acid	290.88
2	Protocatechuic acid	69.61
3	Catechin	248.37
4	Caffeic acid	868.34
5	4-Coumaric acid	2170.78
6	Ferulic acid	691.84
7	Quercetin	1070.33
8	Naringenin	504.65
9	Apigenin	2597.77
10	Caffeic acid phenethyl ester (CAPE)	9153

## Supplementary Materials

The following are available online at https://www.mdpi.com/2072-6643/12/12/3790/s1, Figure S1: Distribution of binding scores.
