# Supplementary material for: Propolis Suppresses UV-Induced Photoaging in Human Skin through Directly Targeting Phosphoinositide 3-Kinase
Source: Nutrients. 2020 Dec 10;12(12):3790. doi: 10.3390/nu12123790 (PMC7764066; doi:10.3390/nu12123790)
Supplement: Supplementary file 1 [file nutrients-12-03790-s001.pdf]

nutrients

MDPI

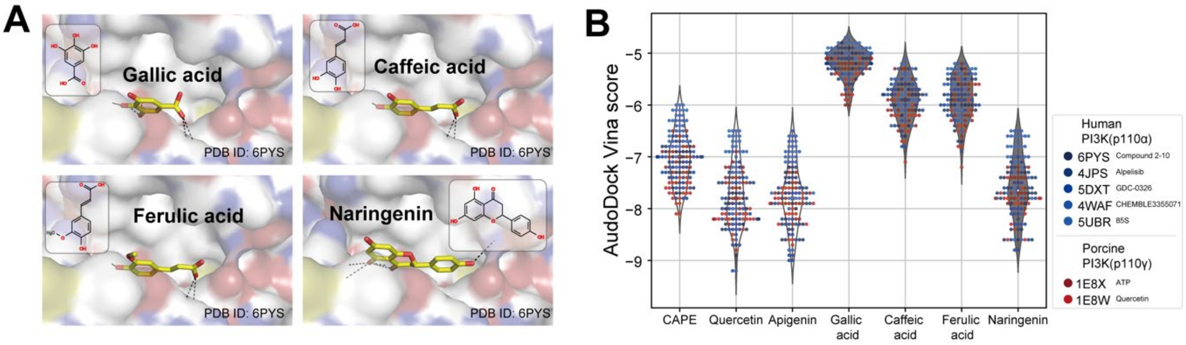

Figure S1. Distribution of binding scores. (**A**) The structural models of gallic acid, caffeic acid, ferulic acid, and naringenin on PI3K; (**B**) The distribution of binding scores for each compound against different structures of PI3K. The docking simulations were executed with multiple PI3K structures that were co-crystallized with different inhibitors. The protein data bank identifiers of the PI3Ks and the names of co-crystalized molecules are marked in the legend.
